# Redefinition of Park Design Criteria as a Result of Analysis of Well-Being and Soundscape: The Case Study of the Kortowo Park (Poland)

**DOI:** 10.3390/ijerph18062972

**Published:** 2021-03-14

**Authors:** Agnieszka Jaszczak, Ewelina Pochodyła, Katarina Kristianova, Natalia Małkowska, Jan K. Kazak

**Affiliations:** 1 Department of Landscape Architecture, University of Warmia and Mazury, 10-719 Olsztyn, Poland; 2 Department of Water Management and Climatology, University of Warmia and Mazury, 10-719 Olsztyn, Poland; ewelina.pochodyla@uwm.edu.pl; 3 Faculty of Architecture, Slovak University of Technology in Bratislava, Námestie Slobody 19, 812 45 Bratislava, Slovakia; katarina.kristianova@stuba.sk; 4 Dream Garden, 10-089 Olsztyn, Poland; natalamalkowska@gmail.com; 5 Institute of Spatial Management, Wrocław University of Environmental and Life Sciences, 50-357 Wrocław, Poland; jan.kazak@upwr.edu.pl

**Keywords:** green therapy, well-being, soundscape, campus park, green university, landscape design, green infrastructure

## Abstract

Well-being and soundscape analysis should be useful in re-design works involved in the planning of recreational areas and green spaces on campuses to improve the health of students, academics, and university staff. Proper space planning, for example, in campus parks, including the planning of soundscape zones, is important in relieving study and work stress. The aim of the study was to conduct a multicriteria analysis of the soundscape and well-being of users in the university park on campus in Olsztyn (Poland). It was important to redefine thinking about the re-composition of park space, including reduction of noise and improving well-being. The research included: 1. measurements of sound pressure levels (SPL) at selected points in two periods, 2. interview with park users and preparation of a mental map, 3. experts’ opinion on soundscape and well-being, and 4. design schemes for re-design of the park. The results of research regarding the perception of the soundscape and well-being in Kortowo park by respondents differ slightly from the results of SPL measurements. The results also confirm the difference between SPL in the leafless and leafy period. The results show a clear relationship between the perception of sounds and well-being in the park. However, in some areas near the water, where higher noise levels are reported the respondents felt quite comfortable. Finally, design schemes are proposed, based on experts’ opinions and results of the analysis.

## 1. Introduction

One of the most important issues while designing green areas in cities, apart from ecological factors and introducing various functions into these areas, is to improve the physical and mental health of their users by more sustainable urban design [1,2]. Such a design approach results from the need to define the correct environment–human relationship, in which it is important to improve ecological structures and appropriate, sustainable use of environmental resources, as well as incorporate education to a broader community [3]. Due to the high importance of that issue urban greenery should be managed with the use of modern technologies and analytical analyses [4].

Among the numerous studies on the planning of recreational areas in the city in a pro-health sense, there are issues related to green therapy, hortitherapy [5], well-being [6], biophilia [7], and restorative effects of the natural environment [8,9,10]. The research domain of soundscape is also becoming more and more important, as evidenced by numerous studies relating to communication spaces [11,12,13,14], urban [15,16,17,18], or suburban built environment [19,20,21,22]. On the other hand, there are fewer studies on methods for minimizing the harmful effects of noise caused by human activity (e.g., transportation) in relation to parks and urban green areas [23], which potentially can improve the well-being of residents. There are only a few studies concerning research on the analysis of the degree of noise nuisance and the improvement of the conditions of use of parks located near educational centers and university campuses [24,25,26]. What is more, research on well-being and soundscape are used to a minimal extent in design practice, especially in the design of parks, which is undoubtedly a major failure [27]. According to the authors, such analyzes should be included in the design practice in the cases of planning new or the re-composition of existing green areas.

Based on the above in this article, we present the procedure aimed at determining the usefulness of multi-criteria pre-design analyzes that could be used in practice. The proposed approach was verified at the university campus, in the Kortowo park (Olsztyn, Poland). The research included measurements of sound pressure levels (SPL) at selected points in two periods. We also interviewed users about the perception of the park in terms of well-being and soundscape, and as a result, we prepared a mental map. Then, we collected the opinion of experts on the issues of designing the park space concerning the well-being and soundscape. The final step was to propose design schemes for the areas that need intervention. First of all, it was important to underline the need to recompose the park, taking into account noise reduction and the improvement of the well-being of its users, i.e., students, scientists, and university employees. The re-composition included the re-planning of four zones in the park selected as a result of earlier analyzes in order to improve well-being and relieve stress related to work and study.

The paper is structured as follows: Section 2 includes literature review; Section 3 describes methods that were applied in the research and the materials that were used to perform the analyses; Section 4 contains results of the research presented in diagrams; discussion and conclusions of the obtained results are presented in Section 5 and Section 6.

## 2. Literature Review

### 2.1. ”Green and Blue” Therapy in Parks

In the era of rapid changes in urban spaces, people need an oasis to rest, relax, or improve well-being [28,29]. Therapeutic effects of greenery and water in parks affect both the physical and psychological conditions of humans. Many authors point to a particular “green and blue” therapy in improving health [30,31,32,33,34,35]. This direction corresponds with Attention Restoration Theory (ART) prosed in 1980s [8,9] which claims that people can concentrate better after spending time in nature or at least after visual perception of scenes of nature. However, as Nghiem et al. [36] noticed there is a need to guarantee a proper ratio between green areas and the number of people spending time there, as the crowdedness limits the positive impact of spending time outdoors. The ART has been also a topic of research conducted in the case of academic campuses; however, Falsten focused his research only on visual perception of natural scenery [37]. At the same time, some researchers highlight that ART lacks adequately tested proves for the strict relationship between exposure to nature and recovery effects and suggest that this domain needs more scientific support to verify the theory [38]. The state of mind and the compassion of the environment in a metaphysical dream are also important. Properly planned parks are both places for rest and contemplation, as well as improve concentration, “refresh” the mind, and most of all allow to increase physical fitness [39,40,41]. Therefore, more and more attention is paid not only to the natural, landscape, or ornamental role of parks and gardens but also to their therapeutic importance. Green therapy is more often included in the development programs of regions with high natural potential and is widely used in spas and health centers [42]. It is also of interest to people involved in the design of landscape architecture objects and planners who adapt the space of parks in resorts, hospital centers, to the requirements of people with various physical or mental abilities [35,43,44,45]. On the other hand, the lack of access to green areas, including parks, may increase the stress caused by work and everyday duties [46].

Referring to the issue of the acoustics of urban space, city parks, and the acoustic properties of greenery and water play an important role in mitigating the effects of noise [47]. When it comes to the results of research on the effectiveness of vegetation in noise reduction, the opinions of researchers vary [48]. Differences between the opinion of park users and measurements by scientists probably result from the psychological perception of greenery and water by users who treat these elements as positive [49,50,51]. The environmental functions of urban parks to improve the soundscape of the urban environment by reducing noise may also be limited by the size and location of city parks [52]. Nevertheless, city parks are considered to be the most important public spaces for the sustainable urban environment, providing users with improved well-being [53,54,55,56]. These are areas that perform regenerative functions [57,58,59].

### 2.2. Designing Green Areas Based on Criteria of Well-Being and Soundscape Perception

Along with the rapid changes in the urban space, including green areas, more research is needed in the direction of an alternative approach to design based on well-being and soundscape. The assumption of planning green areas based on these two factors is to take a comprehensive approach to pre-design analysis issues and implement them at the final design stage. Moreover, poorly designed green areas may not bring the intended effects in improving well-being [60,61].

The method of mapping physical activity in parks, which is one of the well-being factors, was described by Wang and Wu [62] and Hamilton [63]. They investigated the ways of activity in parks depending on the perception of the features and attributes of the environment. According to their approach, factors such as the context of the neighborhood, types of park features, and the state of development, as well as perception may affect the use of the park and the level of well-being. These factors should be the goal of scientists and planners who aim to promote more user-friendly and active parks [64,65].

The need for multidirectional and alternative space planning is emphasized by Aletta and Kang [66], stating that both a better quality of life and a soundscape should be ensured. According to them, it is important to implement new methods of analysis, for example, the perception of environmental sounds and the way in which experiencing them may affect health effects.

### 2.3. Designing Recreational Areas in University Campuses

The design of university campuses varies depending on the scale of each object. Betrabet et al. [67] have analyzed few university campuses in Turkey and the U.S. which significantly differentiated in their sizes. The smallest campus had 14 ha while the biggest one was almost 846 ha. That may influence distinguishing separate zone predesigned for concrete functions. Their study did not take into account soundscapes or other sensory aspects of each campus which may impact perceptions of students. In addition to an aspect of the scale, there are also different approaches to design academic campuses in terms of their concentration. Marrone et al. [68] analyzed that factor at selected European campuses. Their analysis showed that urban compact campuses are in the majority in France (83%), Germany (83%), Spain (74%), or the UK (71%), while most urban campuses in Italy were rather diffused (82%). Both size and concentration of each campus influence the final sustainable performance of a campus. The term green campus can refer to many aspects. Zhu and Dewancker used 17 categories to assess the suitability level of academic campuses, and they took under consideration the engagement of users in social life and operations or processes that are happening in university activity, but among others, they considered also design and planning factors which include the location of greenery and its amount [69]. The importance of that domain can be confirmed by the need for systematic approach creation recently proposed by German researchers. Follmer et al. [70] proposed the Healthy Academic Greenspace Framework, where “campus green spaces need to be considered as spaces for recovery, recreation, interaction and activity which affect students‘ well-being through symbolic, identity-creating, social, cognitive and emotional experiences” which integrates physical, mental and social well-being. In terms of outdoor spaces in university campuses, there are even some guidelines on how to design their spaces as more friendly for sustainable modes of mobility like cycling [71] or walking [72]. Proper design of all these elements can result in a synergy effect improving well-being and soundscape that is perceived as pleasant.

## 3. Materials and Methods

### 3.1. The Study Area

**Figure 1:**
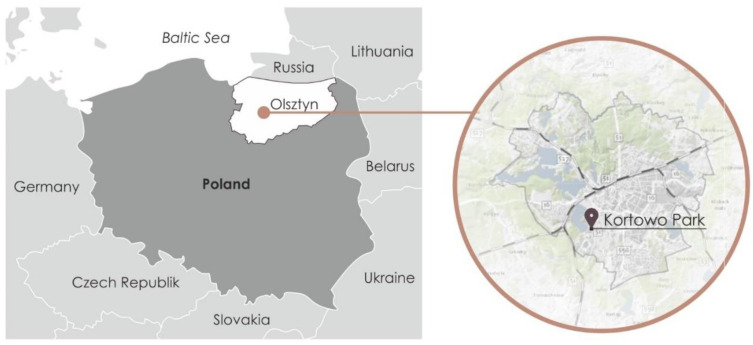
Map with the location of Olsztyn and Kortowo park.

**Figure 2:**
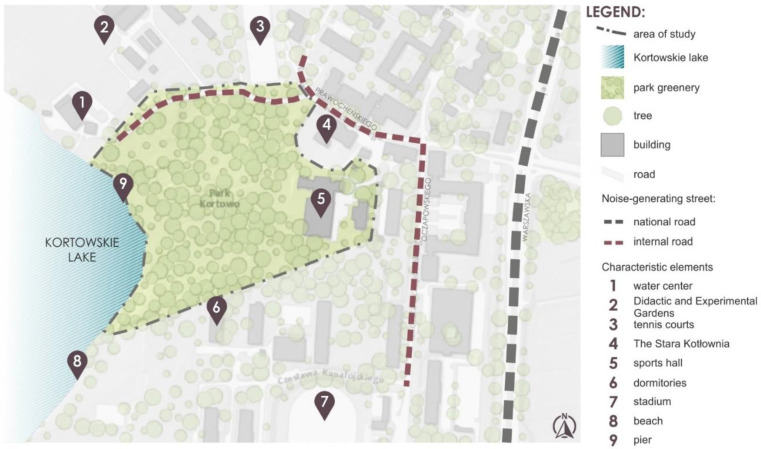
Map with the location of Kortowo park and its surroundings in Kortowo Campus.

The area of study concerns Park Kortowo located in Olsztyn in the Warmia and Mazury Voivodeship (Figure 1 and Figure 2).

The selection of the park for analysis was based on:

- Location—the park is located in the South-West part of Olsztyn near the main national road No. 51 in the direction towards Warsaw
- Accessibility—the park is used every day throughout the academic year by the students, academic staff, and residents and is publicly available
- Source of noise—the park was selected for analysis given the assumption that the main source of noise is generated by transport, especially at the park boundary.
- Functional program—the park has a diversified functional program, in which the leading function is a sport and active and passive recreation. The park has also a historical and aesthetic function.
- Structure of vegetation—is differentiated based on compact plantings of trees and shrubs and large fragments of lawns along with low vegetation.

The basic characteristic of the analyzed park is given in Table 1.

### 3.2. Methodological Steps of Research

**Figure 3:**
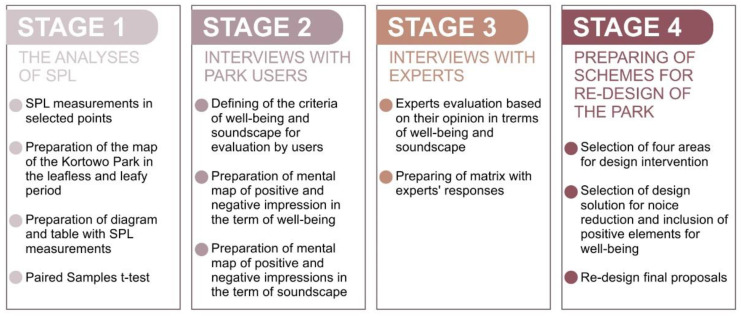
Main stages of the research.

The research was divided into four stages: the analyses of SPL, interviews with park users, interviews with experts, and preparing of schemes for re-design of the park (Figure 3).

The research consisted of an analysis of noise level by SPL measurements in the leafless and leafy periods. It was supplemented by interviews with park users and later with experts. The aforementioned analyses were preceded by a simplified functional and spatial analysis of the park surroundings. The preliminary recognition of the study area, photo inventory, and description of the elements that may affect the soundscape (vegetation, topography, usable zones, street furniture, etc.) were conducted in October 2017. The analyses of SPL measurements were carried out in April and May 2018 in two periods, in leafless and leafy state of vegetation. First, analyses in a leafless state were carried out, and then, in a leafy state. The examinations were performed in the afternoon (2–4 p.m.), during working days (9 April 2018 and 24 May 2018) in moderate wind and sunny weather. There are 28 numbers of characteristics points selected for SPL (Annex 1).

Interviews with users were conducted in the leafless period on purpose, due to the fact that it is during this period that the campus area and the park are used the most (the academic year runs from October to mid-June).

#### 3.2.1. Evaluation of the Soundscape of the Park by the Authors in Selected Points in Terms of SPL

The analyses of SPL aimed at determining which places in the park are most exposed to noise. SPL as A-weighted continuous equivalent sound level (LAeq) were measured in dBA values at nodal points of communication routes and in characteristic places. To achieve reliable results, three measurements were carried out at each place and then averaged. Measurements were taken 1.2 m above the ground (reflecting the fact that park users do not only walk but also sit in the park). The intervals between the three measurements lasted 60 s. A Beha Amprobe 93517D sound level meter was used for the measurement. The results are presented in the form of a chart. The obtained data revealed the quietest and loudest places, as well as the average SPL, for the entire park in leafy and leafless periods. The test procedure also aimed at determining which places in the park are most exposed to noise. Selected measurement points were designated throughout the park in characteristic places, from the park’s borders to its interior, to analyze the entire park space, including the interiors used by visitors for a longer period of the day. In this way, the study was not limited to lanes and access roads, particularly those bordering on transport areas, but also included places where users stayed longer in the park.

The analyses performed in two periods aimed at answering the question of whether and to what extent vegetation in a leafless and leafy state reduces noise in the park. The paired *t*-test was applied to estimate the occurrence of a statistically significant difference between average noise levels during leafless and leafy periods in the park (Annex 2)

#### 3.2.2. Interviews with Park Users—Mental Maps

The subjective opinion of park users was taken into account during the research. The purpose of the survey was to indicate how the park users perceive the space depending on 1. well-being; 2. soundscape perception.

The study in the form of a walk and interview with respondents in the park was conducted in the leafy period, on 9 April 2018. Twenty participants joined the study. The participants were aged 20–55. All of them were living or working in Kortowo Campus during that period (students, and academic staff). The respondents were selected randomly from among students and academics walking in the park, and before starting the interview they were asked about the frequency of staying in the park. People participating in activities in the park at least once per day were selected for the interview. This criterion allowed for the assumption that they know the structure of the park.

The procedure of mapping was preceded by explaining to the participants of the study the assessment criteria in terms of both well-being and soundscape. At the very beginning, the participants got acquainted with the elements that they should pay attention to during mapping. The evaluation criteria are presented below.

Criteria of elements influencing well-being:

- related to the visual perception of various forms of greenery and fauna in the park
- related to the possibility of passive rest and relaxation and active recreation in the park
- related to the visual perception of landscape views in the park
- related to the possibility of integration with other people staying in the park

Criteria of elements influencing soundscape:

- related to the perception of sounds caused by the movement of plants (sound of trees, grasses) and sounds of animals (birdsongs)
- related to the perception of the sounds of water in the park
- related to the sounds of traffic on access roads in the immediate vicinity of the park and on the main roads in the university campus
- related to the perception of the sounds of larger groups of people staying at the same time in the park (e.g., mass events, group meetings)

On the first map, the participants were to take into account the places with the largest and the smallest potential in terms of well-being, i.e., those where they feel comfortable and not comfortable. On the second map, the participants marked areas which they perceive as noisy and on the other hand noise-free and positive-sounding spaces.

Each participant had the task of indicating these areas on the maps. The route ran through the points previously marked in stage 1. The obtained data were digitized and then presented in the graphical form of mental maps.

#### 3.2.3. Interviews with Experts

The experts’ evaluation aimed to find out the opinions of 20 experts (16 women, 4 men), who professionally represent various fields related to spatial planning, urban planning, green therapy, landscape architecture. When selecting experts, the authors made use of the following criteria:

- knowledge of green planning and well-being,
- knowledge of the functional and spatial structure of the analyzed park,
- expert assessment of issues related to the accessibility of the analyzed park for users,
- expert’s ability to indicate the main sources of noise in the analyzed space.

Experts expressed their opinion on the role of green spaces in the well-being of park users. The interview protocol included 9 issues with a descriptive opinion.

Issues in the interview concerned:

- types of activity and elements affecting the well-being and perception of the soundscape of park users,
- incorporation of forms of greenery and infrastructure improving well-being and reducing noise,
- areas requiring intervention in terms of design (inclusion of calming elements, and on the other hand reducing noise),
- pre-design analyzes taking into account the soundscape in practical application in the process of planning parks and green areas.

Respondents were acquainted with the purpose of the interview and were informed about the research assumptions.

#### 3.2.4. Design Schemes for Re-Design of the Park

Works at this stage result directly from the previous stages. On this basis, the places that required the greatest intervention in terms of rearranging the park’s space, reducing unfavorable factors (in this case noise), and including elements improving well-being were selected. For these places, four diagrams were prepared, which took into account, on the one hand, the need for changes resulting from SPL measurements and the interviews with users, and the suggestions of the expert group.

## 4. Results

### 4.1. Analysis in Kortowo Park

#### 4.1.1. Results of Evaluation of SPL Measurements in the Kortowo Park

**Figure 4:**
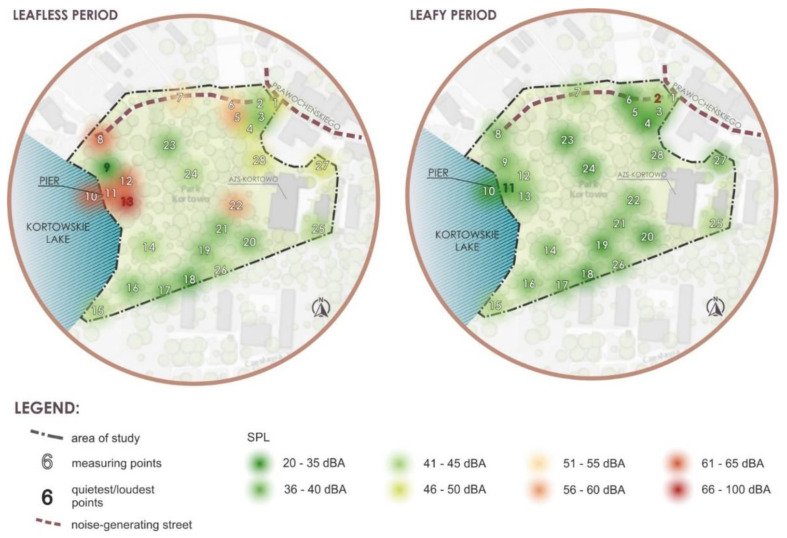
Results of evaluation of the soundscape (SPL measurements) presented in a map of the Kortowo Park in the leafless and leafy period. Traffic is the main source of noise. In the leafless period, point 9 is the quietest, and point 13 is the loudest. In the leafy period, point 11 is the quietest, and point 2 is the loudest.

**Figure 5:**
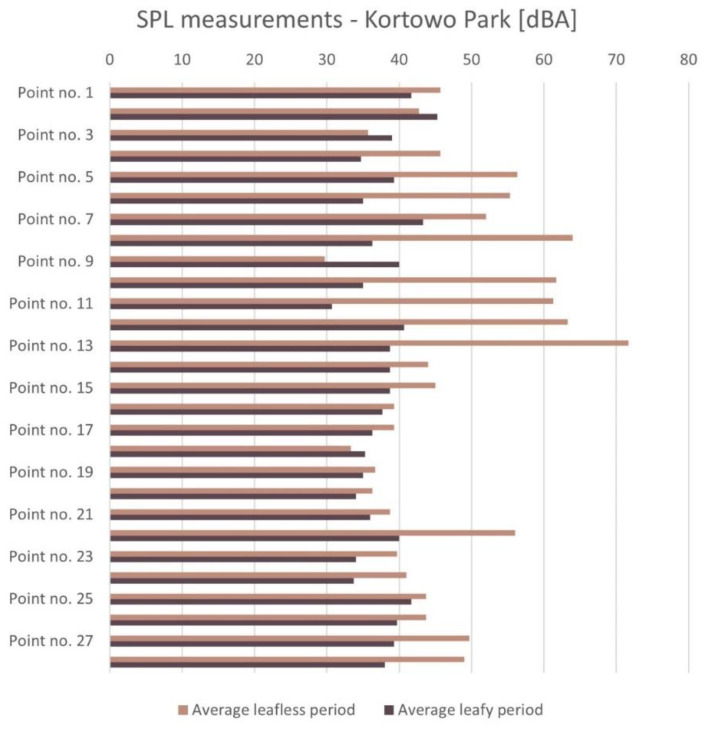
SPL measurements in dBA—Kortowo Park.

In Kortowo Park, 28 measuring points were determined for SPL measurements. The results of SPL measurements are presented in Figure 4 and Figure 5 and Table S1.

Point 9 is perceived as the quietest and point 13 as the loudest in the leafless period. In the leafy period, point 11 is the quietest, and point 2 is the loudest (Figure 4 and Figure 5). It can be stated that in the leafless period the loudest area was located at and on the bridge (points 10–13, in the eastern part of the park near the lakeshore), then close to the entrance to the park area from Prawocheńskiego Street (points 1, 6, and 5), as well as, at point 18. These sounds come both from traffic (northern and eastern part) and they are generated by park users staying in large groups in this place during the day (partly in the southern part and western part near the lakeshore). More intensive noise was also recorded in point 22 near the University Sports Centre and on one of the main paths in the park (in the leafless period). In the leafy period, the slightly increased SPL was recorded at Prawocheńskiego Street (points 2–3) and Oczapowskiego Street (point 25). Leafy period studies indicate that SPLs have decreased in all sensitive areas.

Comparing both maps (Figure 4) it can be stated that noise from transportation comes from the south-west directions where the park borders the housing estate. In the western part, the campus borders the heavily frequented national road.

#### 4.1.2. Result of the Paired Samples *t*-Test in the Kortowo Park

Assuming the significance level (α = 0.05), the value of *p* < 0.00001 was obtained as a result of the dependent sample *t*-test. The noise level significantly increased in the leafless period (M = 47.16, SD = 10.59) compared to the leafy period (M = 37.78, SD = 3.28), t(27) = −4.51, *p* = 0.00012. The result is significant at *p* < 0.05 (Table S2).

### 4.2. Interviews with Park Users—Mental Maps

**Figure 6:**
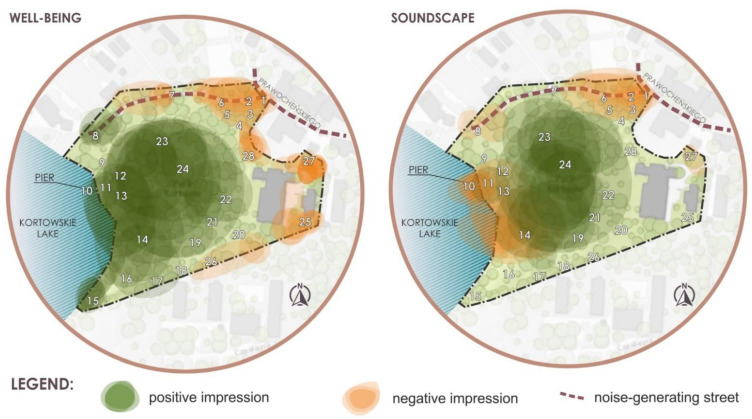
Mental maps of the Kortowo Park with positive and negative impression by park users in terms of well-being and soundscape perception.

According to park users, the most attractive areas in terms of relaxation, and at the same time significantly influencing well-being, are the lakeshore (points 8–15) and in the central part of the park (points 14, 21–23). In the central part, park users identified micro-interiors with tall forms of vegetation, mostly deciduous trees, as places of special importance (Figure 6, well-being).

In terms of soundscape perception they agreed that the noisiest and unpleasant place in the park is the jetty by the lake (points 8–13), the beach (point 14), and the entrance from the Prawocheńskiego Street (points 1–6). Some of the participants identified unpleasant sounds in the area in the central-eastern part, right at the park border (point 27). It is strange, however, that two participants positively assessed the jetty and the beach. The central part of the park turned out to be the highest rated area in terms of sound quality (positive assessment in points 18–26) (Figure 6, soundscape).

### 4.3. The Results of Interview with Experts

**Figure 7:**
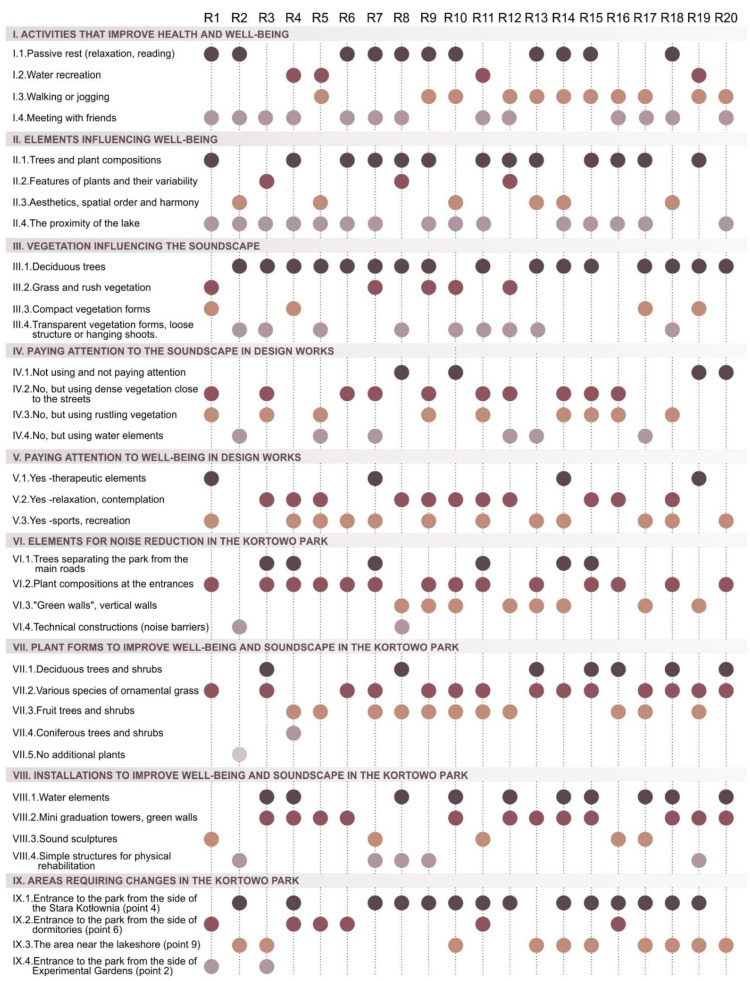
Matrix showing the range of experts’ responses.

According to experts, the improvement of health and well-being of people staying in Kortowo Park is primarily influenced by the following activities: meetings with friends and picnics in open areas, then both walking or jogging along the paths in the park, and passive recreation (relaxing on a bench among greenery). According to them, users practice water sports to a lesser extent (Figure 7, Point I).

“Kortowo Park is primarily a student space. This place is intended mainly for students to relax passively during breaks from university lectures and to socialize... these activities, in my opinion, have the greatest impact on improving health and well-being.”

“Due to the location of the park [university campus], it serves primarily students... the possibility of using numerous paths for active recreation … benches, lawns, platforms for passive rest helps to improve health and well-being.”

Among the elements influencing the perception and thus improving the health and well-being of people staying in the Kortowo Park, experts distinguish the proximity of the lake, the presence of vegetation, especially varied plant compositions. Some of the interviewees believe that the feeling of aesthetics and order, as well as close and distant views (viewing openings) are of great importance. According to them, the features of vegetation (variability, structure) are of less importance (Figure 7, Point II).

“The calming, relaxing power of greenery in combination with the smooth surface of the lake relieves stress and regenerates strength... in my opinion, these factors have the greatest impact on improving the health and well-being of users.”

“...being surrounded by nature, soothing greenery, tall trees [shade, isolation from noise, rustling leaves, a shelter for birds] improve well-being... variability, structure, texture of plants are important, but not the most important... landscape openings, order, and harmony have great meaning, but it seems to me that some of these elements are missing in the Kortowo Park [apart from the lake views].”

When it comes to plant forms that affect the perception of positive sounds of people staying in the Kortowo park, experts indicated primarily deciduous trees, especially those with an openwork habit, a loose structure, species with hanging shoots. Some of the experts also pointed to grass or rush vegetation (Figure 7, Point III).

“The park is dominated by deciduous trees, which definitely influence the perception of positive sounds... of great importance are species with overhanging stems that show potential for pleasant soothing and relaxing humming.”

“It is known that compact vegetation reduces noise, e.g., road noise... but as far as positive sounds in the park are concerned, grass and rushes in windy weather create a pleasant noise which distracts attention from road noise or the buzz of conversation.”

The experts expressed their opinion on their practical use (project preparation) of acoustic maps with noise measurement or soundscape perception analysis. None have used specialized soundscape studies to date, but most try to design dense vegetation along streets adjacent to parks to reduce noise. Some of them introduce leafy vegetation that rustles in the wind or plan water features (cascades, fountains), Figure 7, Point IV.

“I did not know the concept of the soundscape and I have never used any measurement tools when it comes to noise... but I pay attention to shielding busy [noisy] spaces from planned recreational or leisure places in parks.”

“Designing parks is not only taking into account the visual aspects but also creating a friendly environment in terms of acoustics... only in such an environment (visual and sound synergy)... parks can fulfill their role... [if possible] try to choose forms that generate relaxing sounds.”

Experts also referred to the issue of how they relate to the issue of well-being in project practice. It turns out that most often they include recreational and sports elements, as well as therapeutic elements in their park designs. They also try to plan zones for relaxation and contemplation (Figure 7, Point V).

“The primary utility function of the park is to provide a place for recreation, rest and improve the well-being of its users... when designing the park, I try to ensure that each future user can recognize this place as meeting their expectations... I also plan therapeutic and educational elements.”

“I believe that nowadays places for relaxation and contemplation are very desirable and definitely affect the well-being of park space users, therefore I include them in my project.”

Experts propose the following elements that could be introduced to Kortowo Park to reduce noise. Most often they suggest compact plant compositions at the park entrances from the access roads and green walls, elements of the vertical wall from the access roads, and to a lesser extent trees separating the park from the main access roads. According to them, due to the structure and nature of the park, technical noise barriers (i.e., acoustic screens) should not be introduced (Figure 7, Point VI).

“...due to the nature of the park, I would opt for additional plantings in areas polluted with noise [mainly in traffic].”

“...near the access roads in the Kortowo Park, trees appear at a distance [...] but there is no medium-high and low vegetation [...] permanent structures would look unfavorable due to the nature of the site.”

“From the north and east side, I would recommend introducing elements of dense vegetation that would have a double function—entrance zones, inviting and additionally attenuating noise from the adjacent roadways... green walls could appear in the northern part or in places separating the park from the dormitories [south side]... acoustic screens would be a very artificial disfiguring element, therefore I do not recommend them.”

Experts also referred to the introduction of additional vegetation to the Kortowo Park in order to improve well-being and the quality of the soundscape. They propose the use of various species of ornamental grasses (visual and audio effect) and vegetation attracting birds due to bird songs. Some experts also pay attention to deciduous trees and shrubs with a flexible habit (the effect of “rustling” leaves), Figure 7, Point VII.

“The effect of rustling leaves is desirable and I think it would improve well-being and the quality of the soundscape... I suggest including ornamental grasses and creating interesting visual compositions, especially around the lake.”

“The introduction of grasses, especially tall, would allow for an additional effect... ornamental grasses [preferably combined with perennials] can be used to separate interiors that would favor the feeling of isolation, tranquility.”

“...fruiting trees and shrubs are always a good idea... blooming, fruiting [attractiveness at any time of the year], attracting birds [sound and visual effect, especially in winter].”

However, when it comes to the installations that they would introduce to Kortowo Park to improve well-being and the quality of the soundscape, they mention: vertical walls with vegetation, mini graduation towers, water walls (cascades), or, to a lesser extent, sound sculptures (Figure 7, Point VIII).

“Vertical walls have a positive effect on the comfort of being in a given space, soothe emotions, have a calming and relaxing effect.”

“The sound of water is a very pleasant sound... so I propose a vertical wall with a water cascade.”

The experts also pointed to the areas in the park requiring intervention (introducing calming and noise-reducing elements). According to them, a special place that should be re-designed is the area at the entrance to the park on the north side, then the area along the lake, and the border with student dormitories (Figure 6, Point IX).

“It seems to me that the northern part of the park requires intervention... and in the case of strictly noise reduction issues and the introduction of calming elements, I think that the area around Kortowo Lake is a bit too poor... there would be some more elements, e.g., medium and low vegetation, not necessarily trees.”

“...intervention is required in areas near transportation zones where there is intense road traffic.”

### 4.4. Design Schemes for Re-Design of the Park

For formulating management guidelines and guidelines for future design, it becomes crucial to analyze the area in terms of the soundscape, as demonstrated by the results of our research.

**Figure 8:**
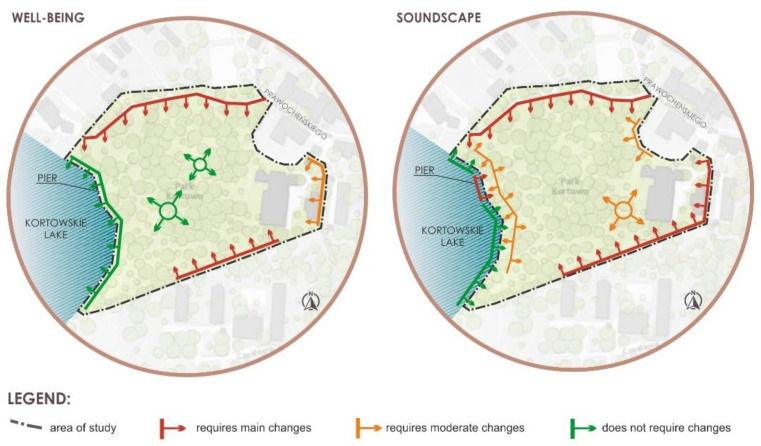
Zones requiring high and medium intervention and zones not requiring changes.

Kortowo Park has a very unstable audio-sphere. This is mainly due to the location on the university campus, on the one hand in the historical part (quiet), and on the other hand in the residential and transportation part (noisy). It should be noted that the park is in the process of revalorization, of the main and historical parts. The overall goal should be to increase biodiversity, and protect flora and fauna, which can also have a positive impact on the soundscape of the analyzed space. However, it is necessary to introduce plants in the form of barriers, especially vegetation, in the recreational area and the lake, as well as near student housing. In addition, experts who previously expressed their opinion on topics related to well-being and soundscape in Kortowo Park, also pointed to the need to re-design certain zones, as shown in Figure 8. These zones cover the northern and south-western parts, as well as a part of the Kortowo Lake shoreline.

Below are proposed changes that we took into account in the concept of re-designing the park, taking into account the issue of improving well-being as well as reducing noise and introducing positive sounds. The proposed vegetation is based on the local habitat.

**Figure 9:**
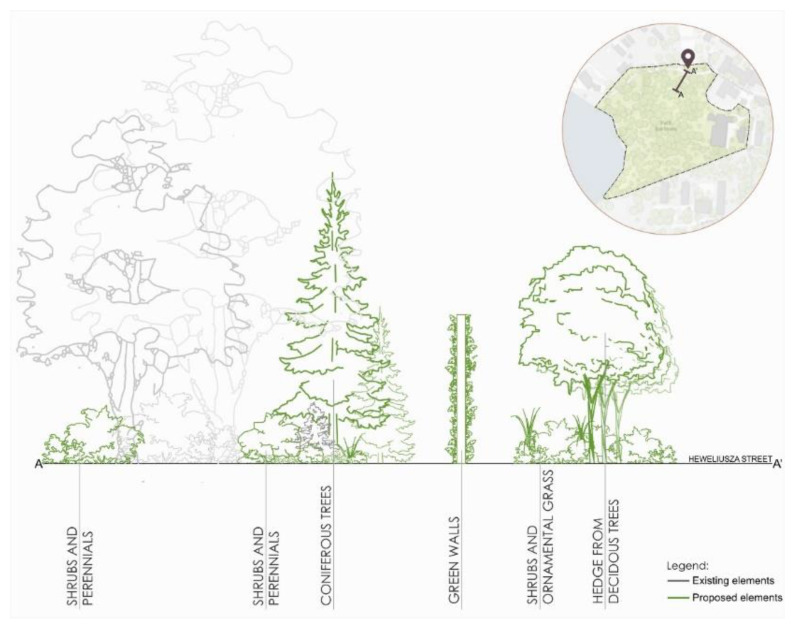
Scheme for re-design of the A zone in Kortowo Park.

The first area is the zone in the northern part of the park, right next to its entrance to the Center of Innovation in Stara Kotłownia. The new concept proposed the introduction of tall deciduous trees (hedges) and compositions of medium-sized ornamental shrubs and grasses forming a barrier in the first line from Heweliusza Street. The second line includes green walls on both sides of the path. The third line includes lower shrubs and perennials emphasizing the park’s naturalistic style and influencing well-being (Figure 9).

**Figure 10:**
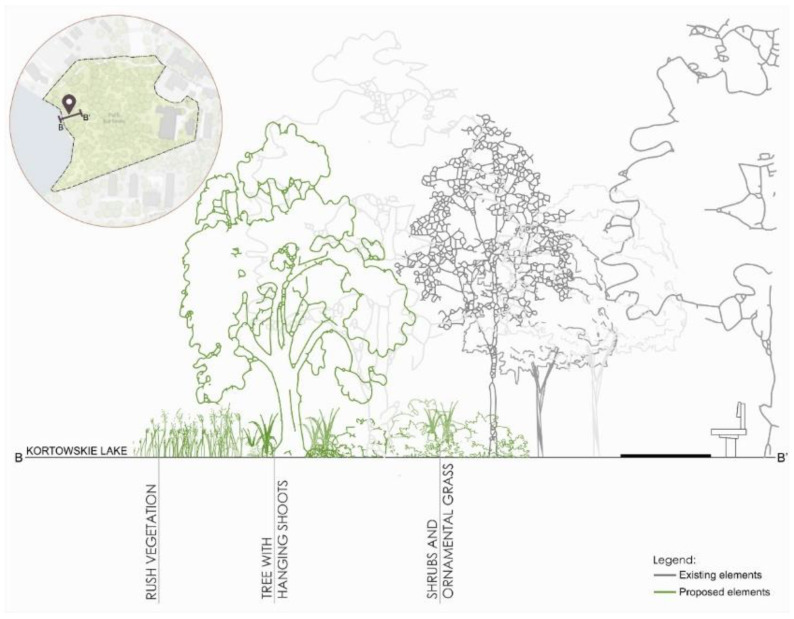
Scheme for re-design of the B zone in Kortowo park.

The second zone is a fragment of the shoreline zone of Kortowo Lake. In this part, it is proposed to enrich with species of rush vegetation the first line of the shoreline. In the second line, trees with drooping shoots and rustling leaves are planned. These trees have an openwork habit due to the necessity to leave a view of the lake from the inside of the park. In the next line, up to the resting area (by the bench), there should appear low shrubs and ornamental trees with rustling leaves (Figure 10).

**Figure 11:**
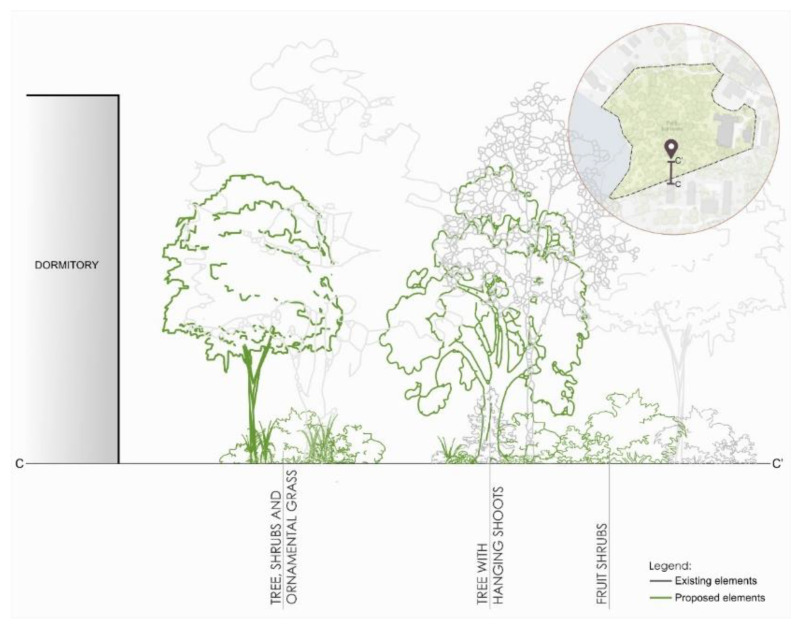
Scheme for re-design of the C zone in Kortowo park.

The third area is the part that forms the border between the park and adjacent students’ dormitories development (southern side). Here, it was proposed to compose tall deciduous trees as well as ornamental shrubs and grasses in the first line from the dormitories. In the next line, trees with drooping shoots are planned, and in the third line of fruiting shrubs (Figure 11).

**Figure 12:**
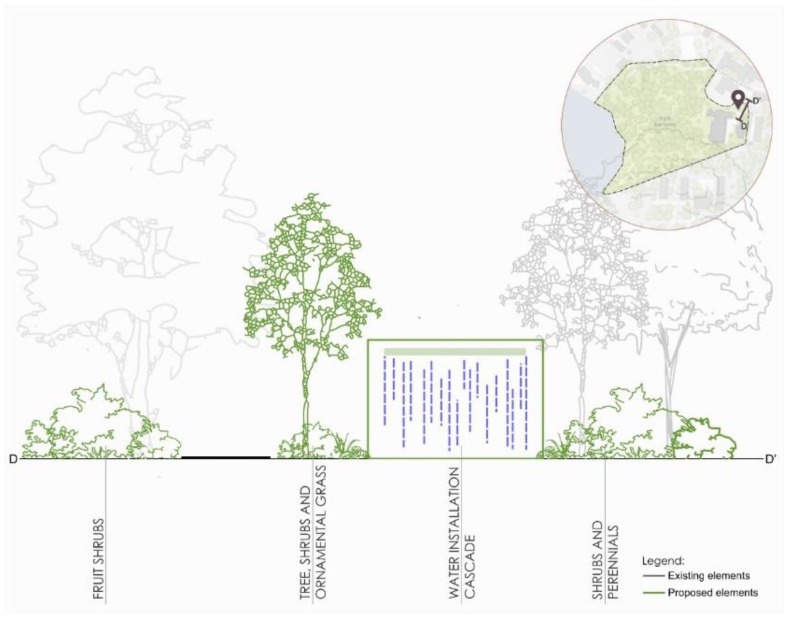
Scheme for re-design of the D zone in Kortowo park.

In the fourth zone, located in the eastern part, behind the sports hall, flowering and fruiting bushes are arranged in the first line (right at the entrance to the path). The second line offers a composition of trees, shrubs, and ornamental grasses of various shapes. A water installation (cascade) is planned just behind the composition. The fourth line includes a composition of deciduous and coniferous shrubs and perennials (Figure 12).

## 5. Discussion

In addition to acoustic valor of greenery in university campuses that is marked in our research, it should be considered as one element in a more complex environmental system, where soundscape together with water management [73], energy consumption [74], or climate mitigation actions [75] can create academic campuses as sustainable educations hubs influencing the surrounding and promoting a built environment of 21st century. Such actions in redesigning academic campuses according to modern urbanization concepts can be observed in many countries around the globe like France [76], Spain [77], Italy [78], Poland [79], Germany [70], Finland [80], US [81], Turkey [82], India [83], China [84], Singapore [85], Malaysia [86], or Brazil [87].

The analyzed park on the university campus has a unique structure. Historical elements like a large part of old trees overlap with modern elements mostly related to the recreational use of this area. Regarding the sources of noise and its intensity, compared to other parks in Olsztyn [88], in Kortowo Park traffic noise is not felt so much due to the considerable distance of the park from the main road. Despite this, along the main access roads to the campus, the impact of noise on the worse well-being of users can be observed. It was shown by SPL measurements in the leafless period and it was confirmed by an interview with users and an interview with experts. According to analyses carried out during the soundwalk interviews in the park, users perceived the park’s area as the least friendly, loud, and annoying in parts which usually border the main road. Similar results were obtained in academic campuses in Turkey [82], India [83], or Brazil [87].

The degree of negative feelings, declared by people assessing the study area, decreases proportionally to the distance from the main road to the interior of the park. It should also be noted that the most positively assessed places are located centrally, away from sources of road noise. Similar patterns were noticed by Mancini et al. [24] or Zannin et al. [89]. There are however a few exceptions. Places with vegetation, especially deciduous trees, were very positively assessed by people participating in our study (interview with users), which may be related to the perception of the sound of trees (leaves) in the wind and the singing of birds. As an acoustically friendly and positive for well-being place in this respect, for example, they distinguished the central part of Kortowo Park. Another positive factor was the water occurrence. Similar results we can observe when analyzing the significance of the soundscape near trees and water, as well as the singing of birds in the perception of users of the assessed area. The importance of perceiving sounds influenced by geophony (sounds of wind, rain, water flow, leaf rustle, etc.) or biophony (sounds of animal vocalizations) is commonly discussed in the research domain [90,91,92,93,94]. On the other hand, the research results showed some contradiction in the case of the lakeshore area assessment. Both the results of the SPL analysis in the leafless state and the interview with park users confirm that the shoreline is the zone with high noise pollution. Despite the typically recreational and resting function of these places and undoubted landscape values, participants are disturbed by noise in this case generated by other users. However, in the assessment of the impact of this area on users’ well-being, an opposite situation can be observed. The lake coastal zone and the central part of the park have been assessed as the most conducive to well-being. So, we can see a certain inconsistency here, which could be caused by the predominance of visual assessment over auditory perception. Researchers are not consistent in opinions when it comes to the major factor influencing the final perception of the well-being of users, considering the role of acoustic and visual factors [95,96,97]. Moreover, they include also thermal conditions as a driver influencing final evaluation [95,96,98,99,100].

Considering the above, while re-designing the park, it is necessary to take into account not only issues related to noise reduction or the introduction of positive sounds but also to implement measures that improve well-being. So we decided to include additional plant forms, but mainly with an openwork structure, in our proposal shown in the design schemes. Therefore, it was not advisable to artificially close the park’s area from the lakeside with a tight barrier only to reduce noise, as it would probably have the opposite effect to the intended one. Experts also pay attention to this issue, suggesting the use of diverse vegetation, including deciduous trees and shrubs of openwork habit, ornamental grasses, and perennials. They propose more compact plant forms at the main entrances to the park. As other authors suggest wide and varied green belts are indicated at the areas near main roads [101,102,103,104,105,106] or in the case of parks between road and entrances to green areas. It is worth introducing in these locations conifers. In our research experts in these zones also proposed other elements, such as green walls or rows of trees. According to the conducted research (an interview with users and an interview with experts), desirable elements to improve well-being in the park are forms of various structures, species attracting birds and insects, water and therapeutic installations introduced in the northern, eastern and south-eastern part of Kortowo Park. These areas partly overlap with those marked as the most heavily burdened with noise (SPL measurements and interview with users). Therefore, in these places, we proposed, among others, appropriate plant compositions, including flowering and fruit forms, forms with rustling leaves, hanging shoots (all designed places), as well as an acoustic screen (fourth scheme). The approach proposed in this study might enrich the current activities of local governments in the field of urban re-development and adaptation to climate change [107,108,109] by supporting architects and planners with more holistic methods for the design of parks and urban public spaces. Currently, the role of parks in human well-being may be even higher due to restrictions connected with the COVID-19 pandemic [110] and anxiety among society due to sedentary behaviors in new daily routines [111].

## 6. Conclusions

The objective of this study was to analyze the well-being and soundscape of Kortowo Park as a part of the landscape planning process to determine the directions of re-design of the place including reduction of noise and improving well-being. The main conclusions are as follows:

- Regarding the spatial and functional structure of the university campus in Olsztyn and the surroundings of the park, a direct relationship exists between the location of the park, functions of surroundings, and noise sources and these factors influence the well-being of park users. It was observed by authors and proved by SPL measurements. It was also confirmed by experts in terms of well-being.
- As confirmed by the conducted *t*-test, the results of SPL measurements in the leafless and leafy period showed that vegetation in the leafless period contributed to noise inhibition less than vegetation in the leafy period.
- The analysis of mental map (interview with users) and answers of experts showed similar results as SPL measurements. These analyses indicated a high risk of noise, especially close to the external borders of the park and along the lakeshore zone. Interestingly, in the case of the perception of well-being in the lakeshore zone, the assessments are opposite, with the lake zone being the most attractive for users.
- There is a relation between the visual and sound perception of the park from a psychological point of view, as confirmed by the results of interviews with park users. The positive impact of vegetation and the proximity of water on the general perception of the park by its users was observed. The visual values contribute to a better perception even though the noise level remains the same.
- Based on the results of the earlier stages of analyzes and interviews, proposals for changes in places requiring intervention were prepared. The proposed changes include the introduction of plant forms with a varied structure and a green wall, a water curtain, not only to reduce noise, but also to improve the well-being and mental and physical condition of campus users.
- The procedure of pre-design works presented in this study is a proposal to include this type of analysis in the design of green areas, particularly parks in university campuses.
- According to the interviews with experts, they do not take into account such methods while designing green areas. Such analyzes may be helpful, especially in the case of designing areas of a therapeutic nature and improving the well-being of users, as well as re-arranging selected parts in existing parks.

The contribution of the presented analyses to the knowledge relates to the use of a simple, multi-criteria analysis process to identify and evaluate the well-being and soundscape of parks in middle-sized Central and Eastern European cities as an element of the design and planning. Importantly, while there are more studies on the topic of well-being and soundscape of parks in the world, there is a lack of research from Central and Eastern Europe, and especially those including a multidimensional approach towards well-being and soundscape. Different geographic, spatial and climate conditions of these regions should be emphasized, which may allow for comparison with other regions in the world.

## Figures and Tables

**Table 1:** The basic characteristics of the examined park.

Park	Location in the City	Total Area (ha)	Forms of Greenery	Type	Special Elements in Parks
Kortowo	Southern part of the center	8	The park is overgrown with old trees, also exotic ones, groups of trees, alleys, perennials	Historical and university park from the beginning of the 19th century	Leisure, recreation, and educational elements, recreational infrastructure at the lake

## Supplementary Materials

The following are available online at https://www.mdpi.com/1660-4601/18/6/2972/s1, Table S1. SPL measurements in selected points, Table S2. Results of the Paired Samples *t*-Test in the Kortowo Park
