# Supplementary material for: Redefinition of Park Design Criteria as a Result of Analysis of Well-Being and Soundscape: The Case Study of the Kortowo Park (Poland)
Source: Int J Environ Res Public Health. 2021 Mar 14;18(6):2972. doi: 10.3390/ijerph18062972 (PMC7999615; doi:10.3390/ijerph18062972)
Supplement: Supplementary file 1 [file ijerph-18-02972-s001.pdf]

Table S1. SPL measurements in selected points.

Point No	Leafless period measurements [dBA]				Leafy period measurements [dBA]			
	I	II	III	Average	I	II	III	Average
**1**	43.0	47.0	47.0	45.7	42.0	44.0	39.0	41.7
**2**	36.0	46.0	46.0	42.7	44.0	45.0	47.0	**45.3**
**3**	41.0	33.0	33.0	35.7	40.0	37.0	40.0	39.0
**4**	37.0	50.0	50.0	45.7	35.0	33.0	36.0	34.7
**5**	43.0	63.0	63.0	56.3	40.0	41.0	37.0	39.3
**6**	48.0	59.0	59.0	55.3	38.0	33.0	34.0	35.0
**7**	50.0	53.0	53.0	52.0	42.0	45.0	43.0	43.3
**8**	60.0	66.0	66.0	64.0	35.0	38.0	36.0	36.3
**9**	31.0	29.0	29.0	**29.7**	42.0	37.0	41.0	40.0
**10**	69.0	58.0	58.0	61.7	32.0	39.0	34.0	35.0
**11**	62.0	61.0	61.0	61.3	30.0	30.0	32.0	30.7
**12**	54.0	68.0	68.0	63.3	43.0	41.0	38.0	40.7
**13**	79.0	68.0	68.0	**71.7**	38.0	36.0	42.0	38.7
**14**	46.0	43.0	43.0	44.0	40.0	43.0	33.0	38.7
**15**	37.0	49.0	49.0	45.0	38.0	36.0	42.0	38.7
**16**	36.0	41.0	41.0	39.3	41.0	38.0	34.0	37.7
**17**	44.0	37.0	37.0	39.3	39.0	37.0	33.0	36.3
**18**	34.0	33.0	33.0	33.3	38.0	36.0	32.0	35.3
**19**	36.0	37.0	37.0	36.7	41.0	31.0	33.0	35.0
**20**	41.0	34.0	34.0	36.3	30.0	34.0	38.0	34.0
**21**	40.0	38.0	38.0	38.7	35.0	33.0	40.0	36.0
**22**	48.0	60.0	60.0	56.0	38.0	42.0	40.0	40.0
**23**	41.0	39.0	39.0	39.7	31.0	37.0	34.0	34.0
**24**	43.0	40.0	40.0	41.0	30.0	35.0	36.0	33.7
**25**	43.0	44.0	44.0	43.7	40.0	42.0	43.0	41.7
**26**	45.0	43.0	43.0	43.7	42.0	37.0	40.0	39.7
**27**	47.0	51.0	51.0	49.7	41.0	38.0	39.0	39.3
**28**	51.0	48.0	48.0	49.0	38.0	36.0	40.0	38.0

Table S2. Results of the Paired Samples *t*-Test in the Kortowo Park.

Point no.	Treatment 1. Leafless Period Measurements -Average [dB]	Treatment 2. Leafy Period Measurements -Average [dB]	*Diff* (T2-T1)	*Dev* (Diff-M)	*Sq. Dev*
1	45.7	41.7	−4	5.38	28.97
2	42.7	45.3	2.6	11.98	143.57
3	35.7	39	3.3	12.68	160.84
4	45.7	34.7	−11	−1.62	2.62
5	56.3	39.3	−17	−7.62	58.03
6	55.3	35	−20.3	−10.92	119.2
7	52	43.3	−8.7	0.68	0.47
8	64	36.3	−27.7	−18.32	335.54
9	29.7	40	10.3	19.68	387.39
10	61.7	35	−26.7	−17.32	299.91
11	61.3	30.7	−30.6	−21.22	450.2
12	63.3	40.7	−22.6	−13.22	174.71
13	71.7	38.7	−33	−23.62	557.8
14	44	38.7	−5.3	4.08	16.66
15	45	38.7	−6.3	3.08	9.5
16	39.3	37.7	−1.6	7.78	60.56
17	39.3	36.3	−3	6.38	40.73
18	33.3	35.3	2	11.38	129.55
19	36.7	35	−1.7	7.68	59.02
20	36.3	34	−2.3	7.08	50.16
21	38.7	36	−2.7	6.68	44.65
22	56	40	−16	−6.62	43.8
23	39.7	34	−5.7	3.68	13.56
24	41	33.7	−7.3	2.08	4.34
25	43.7	41.7	−2	7.38	54.5
26	43.7	39.7	−4	5.38	28.97
27	49.7	39.3	−10.4	−1.02	1.04
28	49	38	−11	−1.62	2.62
	$M_{T1} = 47.16$ $SD_{T1} = 10.59$	$M_{T2} = 37.78$ $SD_{T2} = 3.28$	M = −9.38		SS = 3278.88
